# Application of the artificial intelligence-assisted World Café teaching model in clinical pharmacology graduate course: a pilot study

**DOI:** 10.3389/fpubh.2026.1805521

**Published:** 2026-04-24

**Authors:** Ying Guo, Lan Chun Zhang, Qiong Li, Yun Xia Xiong, Wei Yan Hu, Hai Yun Luo, Jian Ping Xie, Hao Fei Yu

**Affiliations:** 1 Faculty of Basic Medical Science, Kunming Medical University, Kunming, Yunnan, China; 2 Yunnan Key Laboratory of Pharmacology for Natural Products, Department of Zoology, School of Pharmaceutical Sciences, Yunnan College of Modern Biomedical Industry, Kunming Medical University, Kunming, Yunnan, China; 3 Library, Yunnan Minzu University, Kunming, Yunnan, China

**Keywords:** artificial intelligence, clinical decision-making, clinical pharmacology, formative assessment, medical education, teaching reform, World Café

## Abstract

**Background:**

To investigate the application effectiveness of an artificial intelligence (AI)-assisted World Café teaching model in graduate-level clinical pharmacology course and to evaluate its impact on facilitating students’ acquisition and integration of professional knowledge, developing clinical decision-making competencies, and enriching the teaching-learning experience.

**Methods:**

A pilot study was conducted involving 56 first-year master’s students enrolled in a clinical pharmacology course at Kunming Medical University. Instruction was organized around a complex comprehensive clinical case of depression. Students participated in a structured five-stage seminar using the World Café format, supported by AI tools to facilitate group discussions and learning. A multi-method assessment strategy was employed, integrating a teaching effectiveness perception questionnaire, a specialized knowledge test, and a comparative analysis of AI-generated versus instructor-generated scores on case discussion records.

**Results:**

Student evaluations of the teaching model were favorable, with agreement rates exceeding 90% across all items assessing the learning experience. Mean self-rated scores for clinical decision-making abilities each exceeded 4.0 points. Following the intervention, post-test accuracy on depression etiology knowledge improved significantly (median increase from 60.71 to 78.43%; *p* < 0.01). AI-based scoring of case discussion records demonstrated balanced distributions across five assessment dimensions, and total scores showed high concordance with instructor ratings (*p* > 0.05).

**Conclusion:**

The AI-assisted World Café model effectively promotes the assimilation of complex knowledge and fosters higher-order clinical decision-making abilities among clinical pharmacology graduate students. It facilitates a pedagogical shift from passive knowledge transmission toward active capacity building. Furthermore, AI demonstrated reliability and promising utility as a tool for formative assessment, offering empirical support for innovative human-computer collaborative teaching approaches.

## 1 Introduction

Clinical pharmacology education is a key component in cultivating the core competencies of future medical and pharmacy experts. Its objectives extend beyond imparting theoretical knowledge such as drug mechanisms of action and therapeutic principles; rather, they aim to cultivate students’ decision-making competence in critically analyzing, synthesizing diverse evidence, and formulating individualized treatment plans within complex and uncertain clinical contexts (1, 2). However, traditional instructional models, including teacher-centered lectures or loosely structured case discussions, frequently encounter several challenges. These include uneven student participation, difficulty in exploring pathophysiological mechanisms in depth, the instructor’s limited capacity to provide real-time personalized feedback, and an insufficient simulation of the dynamics and complexity inherent in real-world clinical decision-making (3, 4). Consequently, these methods show significant limitations in fostering essential higher-order cognitive skills, such as critical thinking and clinical reasoning (5).

Recent breakthroughs in artificial intelligence (AI) technology have created unprecedented opportunities for innovation in medical education (6, 7). The integration of AI is widely regarded as a pivotal avenue for addressing pedagogical challenges and advancing active, technology-enhanced learning (7). Evidence suggests that AI can function as a powerful cognitive tool by providing personalized learning resources, simulating clinical dialogues, and assisting with literature synthesis and data analysis (8–10). This capability can help mitigate educational resource constraints and foster more interactive, adaptive learning environments. Currently, large language models (LLMs) such as ChatGPT, DeepSeek, Google Bard, and dedicated AI tools show promising potential in applications like medical information retrieval, case simulation, and learning facilitation (10, 11). Concurrently, collaborative and structured discussion-based pedagogies such as the World Café model are gaining traction in higher medical education, recognized for their effectiveness in promoting profound dialogue, co-construction of knowledge, and integration of multiple perspectives (12, 13). This model fosters an egalitarian and open communicative atmosphere, encourages participant rotation across discussion groups, and facilitates the cross-pollination of ideas and the generation of collective wisdom (14, 15).

Despite the significant individual potential demonstrated by both AI and collaborative learning models, there is a paucity of systematic design and empirical research on their deep integration. Specifically, the incorporation of AI as a cognitive partner and source of real-time scaffolding (16) within structured discussion processes like the World Café remains underexplored. Such integration could create a learning environment that stimulates high-level cognitive engagement while providing intelligent support. This gap persists both in clinical pharmacology and in medical education at large. Current research predominantly focuses on the independent application of AI tools or the evaluation of traditional teaching methods, failing to adequately explore the role of human-computer collaborative learning cycles in fostering students’ advanced clinical decision-making abilities (17).

Therefore, this study aims to apply the AI-assisted World Café model to a complex, comprehensive depression treatment case within a clinical pharmacology graduate course and to evaluate its implementation. This study hypothesizes that this integrated model can: (1) effectively enhance students’ mastery and comprehension of core professional knowledge; (2) significantly improve their self-efficacy and cognitive competencies in clinical decision-making; and (3) leverage AI-assisted process evaluation to provide reliable formative feedback on the quality of group discussions. This study seeks to provide empirical evidence and a practical instructional framework for the deep integration of AI and collaborative learning theory in medical education, addressing the challenges of cultivating outstanding talents competent for future medical practice.

## 2 Methods

### 2.1 Participants

Participants were first-year master’s students enrolled in the clinical pharmacology course during the autumn semester (September 2025 to January 2026) at Kunming Medical University. A total of 56 students were included, with a male-to-female ratio of 20:36 and a median age of 24 years. All participants provided voluntary informed consent. Two questionnaire surveys were administered: the first yielded 56 valid responses, and the second yielded 51. There were no statistically significant differences in gender, age, and interest in learning clinical pharmacology between students participating in the pre-test and post-test (all *p* > 0.05) (Supplementary material 1).

### 2.2 Study design

A pilot design was employed to evaluate the effect of the AI-assisted World Café model in a clinical pharmacology course. The instructional intervention centered on a complex case of depression management. Students were randomly assigned to groups of 5–6. Each group engaged in a five-stage structured seminar addressing three sequentially advanced clinical questions: diagnosis and differential diagnosis, pathological mechanism analysis, and individualized treatment strategy formulation. All groups completed a case discussion record sheet (Supplementary material 2).

The process consisted of five stages. Stage 1 involved groups reviewing the case materials and forming preliminary clinical analyses. In stage 2, each group collaboratively formulated 1–2 open-ended, mechanism-focused questions to pose to a designated LLM to deepen or test hypotheses. During stage 3, members critically evaluated the AI-generated responses based on information accuracy, logical consistency, and potential limitations. Stage 4 required a designated member from each group to rotate to another table, sharing key discussion points, AI-derived insights, evaluation outcomes, and pending questions, while recording new perspectives gained. Finally, in stage 5, all members returned to their original groups, integrating initial hypotheses, AI-generated information, critical evaluations, and insights from other groups to formulate a final treatment plan for presentation. The instructor then provided summative feedback on each group’s discussion process and outputs. Before the session, students received a personalized summary of key concepts related to depression, generated by AI based on their prior knowledge. After the session, each student received a report summarizing their contributions, thought processes, and suggested areas for improvement, generated by AI based on their group discussion records.

Students received training on formulating structured prompts (e.g., Explain the mechanism of…, Compare treatment options for…, What are the risks of…). Although prompts were not strictly standardized across groups to allow for exploratory learning, all groups were required to document their prompts and the corresponding AI responses in the case discussion record sheet. To mitigate the risk of AI hallucinations, students were instructed to critically evaluate AI outputs and cross-check information against trusted sources, including clinical guidelines and textbooks. Instructors also monitored AI responses in real time and intervened when inaccuracies were identified.

### 2.3 Teaching effectiveness evaluation

**Figure 1:**
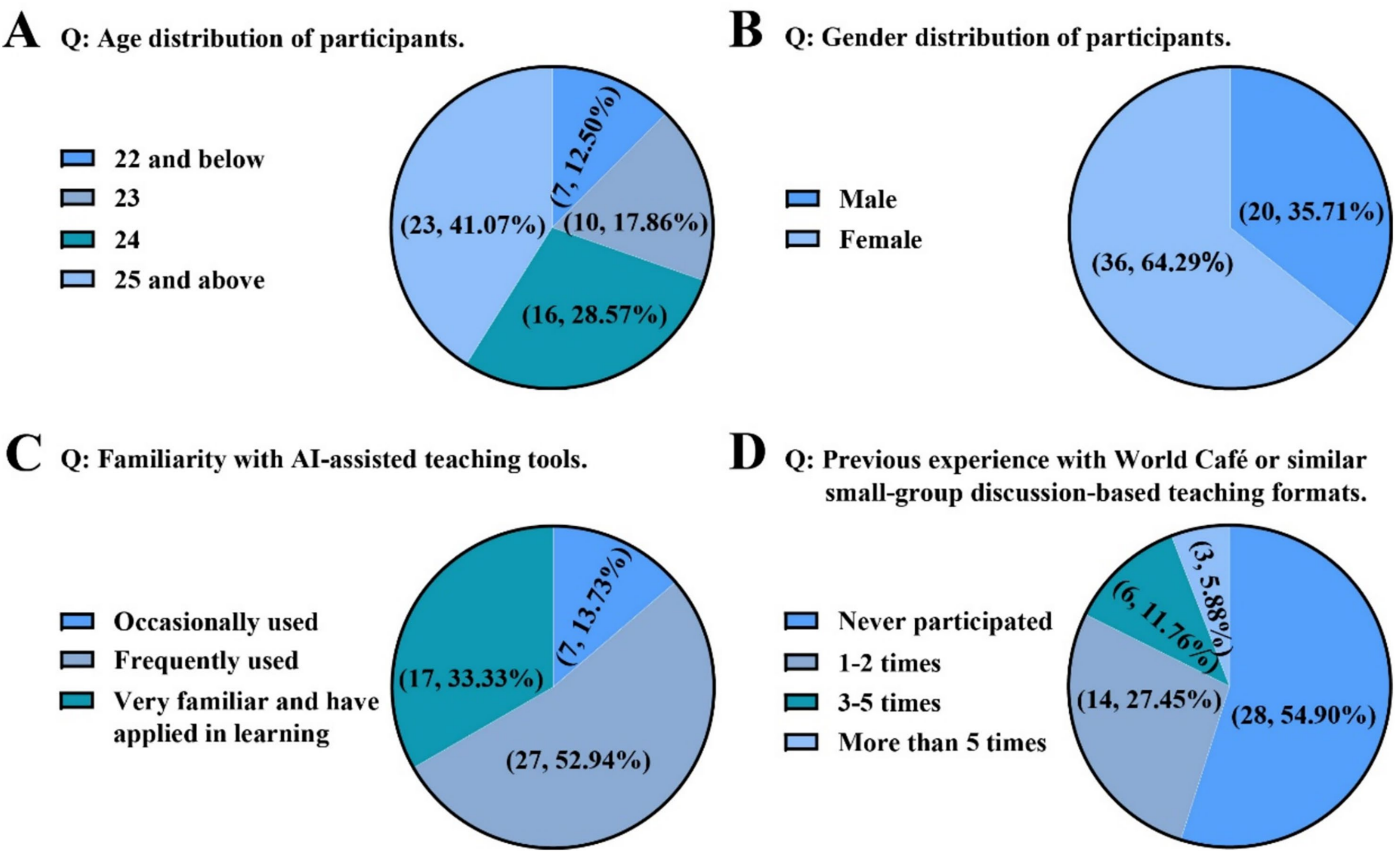
Participants’ basic characteristics distribution. **(A)** Age distribution. **(B)** Gender distribution. **(C)** Familiarity with AI-assisted teaching tools. **(D)** Previous experience with World Café or similar small-group discussion-based teaching formats.

**Figure 2:**
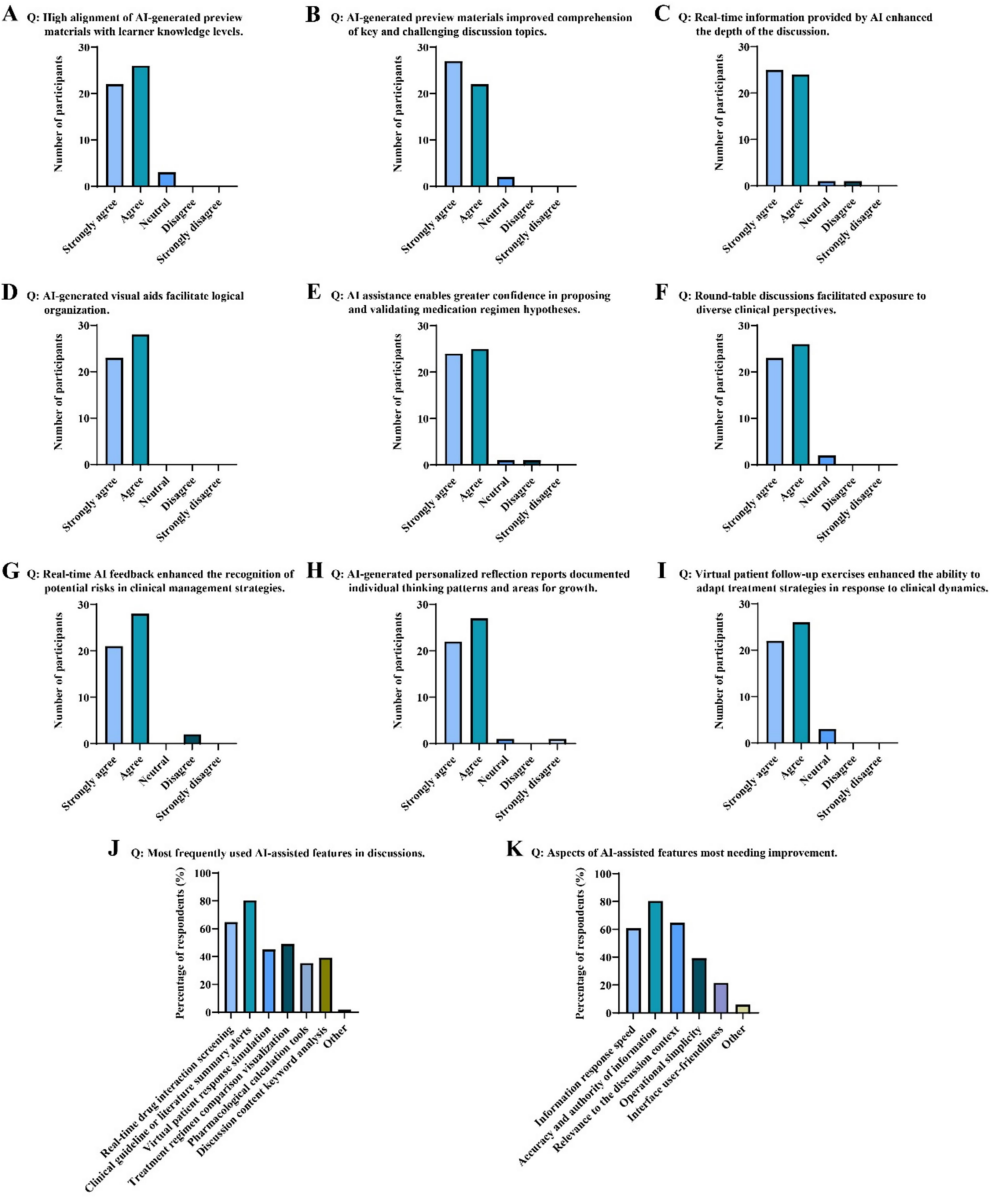
Student evaluation and feedback on the AI-assisted World Café teaching model. **(A)** High alignment of AI-generated preparatory materials with learner knowledge levels. **(B)** AI-generated preview materials improved comprehension of key and challenging discussion topics. **(C)** Real-time information provided by AI enhanced the depth of the discussion. **(D)** AI-generated visual aids facilitate logical organization. **(E)** AI assistance enables greater confidence in proposing and validating medication regimen hypotheses. **(F)** Round-table discussions facilitated exposure to diverse clinical perspectives. **(G)** Real-time AI feedback enhanced the recognition of potential risks in clinical management strategies. **(H)** AI-generated personalized reflection reports documented individual thinking patterns and areas for growth. **(I)** Virtual patient follow-up exercises enhanced the ability to adapt treatment strategies in response to clinical dynamics. **(J)** Most frequently used AI-assisted features in discussions. **(K)** Aspects of AI-assisted features most needing improvement.

**Figure 3:**
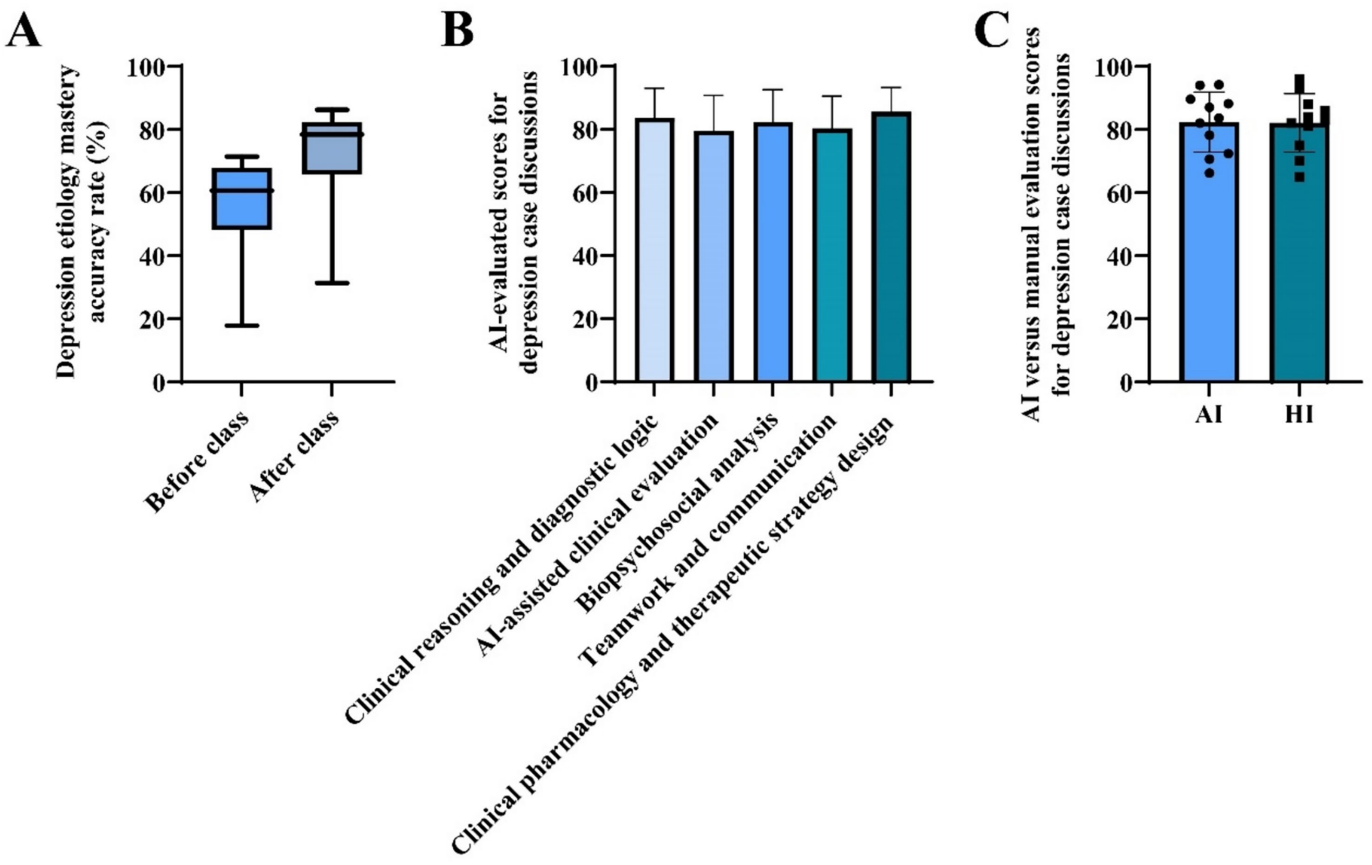
Multidimensional evaluation of learning outcomes in depression case within the AI-assisted World Café teaching model. **(A)** Answer accuracy rates on etiological knowledge of depression before class and after class. **(B)** AI evaluates student group discussions on depression cases from five aspects: clinical reasoning and diagnostic logic; AI-assisted clinical evaluation; biopsychosocial analysis; teamwork and communication; and clinical pharmacology and therapeutic strategy design. Statistical analysis was using a one-way ANOVA. **(C)** Mean scores between AI and human evaluations of student performance in depression case discussions. Data were analyzed using the intraclass correlation coefficient. AI, artificial intelligence; HI, human intelligence.

Teaching effectiveness was evaluated through a multi-source data approach. The questionnaire was developed based on a comprehensive literature review of prior studies on the World Café model, AI in education, and clinical decision-making competencies. The initial item pool was reviewed by three experts in medical education and clinical pharmacology for content validity. A pilot test was conducted with 10 graduate students (not included in the main study) to assess clarity, relevance, and comprehensibility. Based on their feedback, minor wording adjustments were made. The final questionnaire demonstrated good internal consistency, with Cronbach’s *α* = 0.89 for the entire scale. A post-course questionnaire was developed for this study (Supplementary material 3), and was administered to the experimental group. A background questionnaire captured participants’ demographic information, familiarity with AI tools, and prior experience with discussion-based teaching, providing an overview of the sample characteristics (Figure 1). Perceptions of the teaching model were assessed immediately after the session using a three-part questionnaire. The first part measured students’ subjective appraisal of the model’s usefulness on a five-point Likert scale (1 = strongly disagree, 5 = strongly agree). The second part collected feedback on AI tool usage and optimization needs, identifying the most frequently used functions and aspects requiring improvement (Figure 2). The third part consisted of a self-assessment of clinical decision-making abilities, also using a five-point scale to evaluate improvement across five core competencies (Table 1). Knowledge acquisition was measured with an identical standardized test covering 12 core concepts in depression etiology, administered before and after the intervention to quantify learning gains (Table 2, Figure 3A). Process evaluation of the case discussions consisted of two components. AI-based scoring, in which the DeepSeek tool (version DeepSeek-V3.1) analyzed and scored group discussion records across five dimensions (Figure 3B), and manual scoring, where instructors independently rated the same records under blinded conditions (Figure 3C). AI-based and instructor scoring both used the same rubric (Rubric for Evaluating AI-Assisted Discussion Records). This rubric evaluates five dimensions: clinical reasoning and diagnostic logic, AI-assisted clinical evaluation, biopsychosocial analysis, teamwork and communication, and clinical pharmacology and therapeutic strategy design. Each dimension is rated on a four-level scale: 90–100 (excellent), 75–89 (good), 60–74 (fair), and 0–59 (poor). The complete rubric is provided in the Supplementary material 4.

**Table 1:** Student self-assessment of the AI-assisted World Café model in developing clinical decision-making skills.

Questions	Student feedback, %					
	5	4	3	2	1	Mean
Enhances the ability to integrate multi-source evidence for clinical decision-making.	23 (45.10)	25 (49.02)	2 (3.92)	0 (0.00)	1 (1.96)	4.35
Improves the capacity to anticipate and mitigate clinical medication risks.	19 (37.25)	28 (54.90)	2 (3.92)	2 (3.92)	0 (0.00)	4.25
Strengthens the ability to articulate, debate, and refine viewpoints within a team.	18 (35.29)	28 (54.90)	2 (3.92)	3 (5.88)	0 (0.00)	4.20
Builds clinical confidence in managing complex and atypical cases.	19 (37.25)	26 (50.98)	4 (7.84)	2 (3.92)	0 (0.00)	4.22
Advances the competency to translate pharmacological knowledge into personalized therapeutic decisions.	19 (37.25)	24 (47.06)	5 (9.80)	2 (3.92)	1 (1.96)	4.14

**Table 2:** Comparison of professional knowledge mastery in the AI-assisted World Café model for clinical pharmacology.

Questions	Accuracy rate, %	
	Before class	After class
Classical neurobiological hypotheses of depression etiology.	23.21	41.18
The core neuroendocrine hypothesis in depression pathogenesis.	50.00	66.67
The theory of impaired neural plasticity in depression.	57.14	86.27
A macro-level overview of the immune-inflammatory theory of depression.	46.43	64.71
Comprehension of gut–brain axis principles and bidirectional pathways.	71.43	84.31
Specific pathological mechanisms linking gut microbiota to depression.	71.43	78.43
Potential gut–brain axis interventions and their scientific rationale.	64.29	82.35
Clinical and biological evidence linking inflammation to depression.	62.50	82.35
Gut microbiota as a key source of inflammation: an intersecting concept.	71.43	82.35
Mechanistic overlap and bidirectional relationship between antidepressant and anti-inflammatory effects.	51.79	66.67
The role of microglia as central executors of neuroinflammation in depression.	60.71	80.39
Alterations in brain-derived neurotrophic factor in depression and its significance as a therapeutic target.	62.50	70.59

### 2.4 Data analysis

Data were analyzed using GraphPad Prism 9.0. Questionnaire reliability was assessed with Cronbach’s *α*. Categorical data are reported as frequencies and percentages; test scores as medians and percentages; and case evaluation scores as means. The Shapiro–Wilk test checked normality. Due to anonymous data collection, paired-sample *t*-tests were not feasible for answer accuracy rates before class and after class. That results are therefore presented using descriptive statistics. Normally distributed data with homogeneous variance were analyzed with one-way ANOVA (AI dimension scores). To assess the validity of the AI-assisted process evaluation, the intraclass correlation coefficient (ICC) between the AI and human instructor ratings was calculated using a two-way mixed-effects model based on absolute agreement.

## 3 Results

### 3.1 Participant characteristics

A total of 56 participants were included in this study. Their demographic and background characteristics are summarized in Figure 1. The age distribution was as follows: ≤22 (12.50%), 23 (17.86%), 24 (28.57%), and ≥25 (41.07%) (Figure 1A). The cohort comprised 35.71% male and 64.29% female students (Figure 1B). Regarding familiarity with AI-assisted teaching tools, 52.94% of students reported frequent use, and 33.33% reported being very familiar and having used such tools for academic purposes (Figure 1C). However, 54.90% of participants had no prior experience with the World Café or similar structured discussion formats, with only 5.88% having participated more than five times (Figure 1D).

### 3.2 Subjective evaluation and self-assessed competencies

Students’ perceptions of the AI-assisted World Café model were predominantly positive (Figure 2). The agreement rate (combined percentage of “agree” and “strongly agree” responses) exceeded 90% for all evaluation items. In the preview phase, 94.12% of students perceived the AI-generated personalized preview materials as well-aligned with their prior knowledge base (Figure 2A), and 96.08% found these materials helpful for understanding key and challenging concepts for the upcoming discussion (Figure 2B). During the World Café discussion phase, 96.08% agreed that real-time AI-provided information deepened the discussion (Figure 2C); 100% indicated that visual aids helped clarify logical reasoning (Figure 2D); 96.08% reported increased confidence in proposing and validating clinical hypotheses with AI support (Figure 2E); 96.08% agreed that the table rotation exposed them to more diverse clinical perspectives (Figure 2F); and 96.08% believed AI’s instant feedback helped identify potential risks in treatment plans (Figure 2G). In the post-discussion phase, 96.08% felt the AI-generated personalized reflection report accurately captured their thought processes and areas for growth (Figure 2H), and 94.12% believed the virtual patient follow-up task reinforced their understanding of dynamic treatment plan adjustment (Figure 2I). Regarding AI tool utilization and suggested improvements, the most frequently used functions were clinical guideline or literature summarization alerts (80.39%) and real-time drug interaction screening (64.71%) (Figure 2J). Areas most frequently highlighted for improvement concerned information accuracy and authority (80.39%), and contextual relevance to the discussion (64.71%) (Figure 2K).

Analysis of students’ self-assessment regarding clinical decision-making competencies within the AI-assisted World Café model is presented in Table 1. Over 90% of students perceived that, compared to traditional instruction, this model significantly enhanced five core competencies: multi-source evidence integration, anticipation and mitigation of clinical medication risks, elaboration and revision of perspectives within a team, confidence in managing complex cases, and translation and application of pharmacological knowledge. Measured on a five-point Likert scale, the mean scores for all five self-assessment items exceeded 4.0.

### 3.3 Professional knowledge test performance

Test results demonstrated an improvement in students’ accuracy rates on core depression etiology knowledge following the intervention (Table 2). The most substantial gains were observed in knowledge points requiring mechanistic interpretation and integrated application, notably neuroplasticity impairment theory (29.13% improvement). Comparatively smaller improvements were noted for some specific, micro-level descriptive mechanistic knowledge, such as the precise pathological mechanisms by which gut microbiota influences depression (7.00% improvement).

An overall assessment of professional knowledge mastery was conducted. Descriptively, the median accuracy rate increased from 60.71% in the pre-test to 78.43% in the post-test (Figure 3A), indicating that the teaching model effectively promoted students’ comprehension and mastery of the depression etiology knowledge system.

### 3.4 Scoring of depression case discussion records

The AI evaluation of student group discussion records for the depression case showed no significant differences across five assessed dimensions, as determined by one-way ANOVA: clinical reasoning and diagnostic logic, quality and critical appraisal of AI interaction, biopsychosocial comprehensive analysis, teamwork and integrative expression, clinical pharmacological knowledge application and treatment strategy design, with mean scores of 83.73, 79.64, 82.36, 80.36, and 85.64, respectively (all *p* > 0.05, Figure 3B). The AI evaluation and the manual evaluation (HI) yielded mean scores of 82.36 and 82.09, respectively. The average-measure ICC was 0.915 (95% CI: 0.679–0.977), indicating excellent reliability. These findings suggest strong agreement between the AI and HI ratings, supporting the use of their averaged scores as a reliable measure.

## 4 Discussion

This study evaluated the application of a novel instructional model integrating AI with the World Café discussion format in a clinical pharmacology graduate course. Results indicate that the model significantly improved students’ mastery of professional knowledge regarding depression etiology (Figure 3A). It also garnered high ratings in self-assessed clinical decision-making competencies (Table 1). In addition, students positive evaluations of the teaching and learning experience (Figure 2).

Following the intervention, students’ overall accuracy on the depression etiology test increased significantly. However, knowledge gains exhibited a notable structural pattern. The greatest improvement was observed for complex, interdisciplinary knowledge points requiring mechanistic explanation and integration, such as those involving neuroplasticity and inflammatory pathways. In contrast, improvement was more limited for relatively discrete, descriptive factual knowledge. This pattern suggests that the model’s strength lies in fostering conceptual understanding and knowledge transfer, rather than rote memorization. This aligns with the foundational pedagogical aim of the World Café method, which is to promote deep understanding and co-construction of meaning through structured, multi-stage dialogue (13, 15). Within this design, students engaged in an iterative cycle. This included group discussion, AI inquiry, critical appraisal, inter-group rotation, and final synthesis. The process effectively simulated the cognitive dynamics of real-world multidisciplinary team consultations. Crucially, AI functioned as a cognitive load redistributor (18, 19). In traditional discussion settings, students’ cognitive resources are often consumed by fact-checking, which can impede deeper mechanistic exploration. In this study, AI served as an on-demand information source and explanatory tool (20, 21). It handled foundational tasks and thereby freed students’ cognitive capacity. This allowed them to engage in higher-order thinking, including hypothesis generation, evidence appraisal, and therapeutic plan design. This likely explains the pronounced improvement on knowledge points requiring analytical synthesis.

Students reported high self-assessed competencies in areas such as multi-source evidence integration, medication risk anticipation, and confidence in managing complex cases. This perceived growth likely stems from a synergistic effect of technological and social empowerment. Technologically, the AI-provided resources made implicit clinical reasoning explicit. These resources included mechanism diagrams, treatment plan comparisons, and virtual patient follow-ups. They also created a safe, simulated environment for iterative hypothesis-testing-revision practice (22, 23). This approach resonates strongly with experiential learning theory (24, 25). Social empowerment was achieved through the World Café’s rotation mechanism. This mechanism disrupted potential intellectual echo chambers within fixed groups. It exposed students to diverse clinical perspectives, thereby effectively fostering clinical reasoning skills (26). Unlike traditional group discussions that primarily enhance communication and teamwork, this model, by integrating AI as a dynamic cognitive partner for each group, established a human-AI-human triadic interaction. Students were engaged not only in debate with peers but also in a continuous process of citing, questioning, and calibrating against the AI-provided, evidence-informed information. This interaction significantly enhanced the rigor of discussions and the depth of knowledge integration.

However, student feedback also reflected a critical stance toward the technology. Their primary concerns focused on information accuracy and contextual relevance. These highlight core limitations of current general-purpose LLMs in specialized domains. Such limitations include risks of hallucination, knowledge update latency, and deficiencies in contextual understanding (27–29). In the context of clinical education, such limitations pose non-trivial risks. AI-generated content may present fabricated references, plausible yet incorrect pathophysiological explanations, or omit critical patient-specific nuances, potentially leading to cognitive errors if accepted uncritically by learners (30). Unchecked reliance on AI-generated information during clinical reasoning tasks could inadvertently reinforce misconceptions or compromise diagnostic accuracy (31). Recognizing these risks, the instructional design of this study deliberately embedded structured critical appraisal as a mandatory component of the learning workflow. Students were explicitly guided to verify AI outputs against authoritative sources and to evaluate logical consistency and completeness. This approach reframes AI hallucination not merely as a technical flaw to be mitigated, but as a pedagogical opportunity to cultivate essential clinical competencies, such as source criticism, evidence validation, and uncertainty management. This discerning attitude does not constitute technology rejection but rather reflects an emerging and valuable form of critical AI literacy (32, 33). Students demonstrated an understanding that AI output represents a hypothesis requiring professional scrutiny and contextual calibration, not a definitive conclusion. Therefore, the structured critical appraisal of AI-generated responses represents an essential component of this study. This process itself constitutes a high-order clinical reasoning exercise, training students to be prudent evaluators of AI-generated content. This directly addresses calls from the medical education community for integrating AI literacy training (21, 34).

AI-generated scores for group discussion records were distributed evenly across five clinical competency dimensions, and the total score showed high consistency with blinded instructor scoring. This provides preliminary empirical support for exploring human-computer collaborative (17, 35) intelligent formative assessment. It suggests that, when guided by highly structured tasks and explicit evaluation rubrics, AI shows potential in capturing representations of high-order abilities such as clinical reasoning, knowledge application, and teamwork (36, 37). While aligning with trends applying AI to structured assessments like the Objective Structured Clinical Examination (38), this study further demonstrates its applicability in open-ended, natural language discussion contexts. The significance lies in AI’s potential to enable large-scale, personalized, and immediate process feedback (39), which is crucial for facilitating deliberate practice (40) and the timely correction of cognitive biases. It must be emphasized, however, that this consistency does not imply AI can replace instructor expertise (41). This suggests a potential future model of collaborative evaluation, characterized by AI preliminary analysis followed by teacher deep empowerment. Within this framework, AI functions as an efficient initial screener. It performs pattern recognition and first-pass assessment. Teachers subsequently integrate clinical experience, ethical considerations, and personalized guidance to deepen the evaluation. Future research must thoroughly investigate the validity boundaries, fairness (42), and implementation protocols of such a model.

### 4.1 Limitations

The lack of a control group precludes definitive attribution of the observed improvements to the AI-assisted World Café model. Therefore, this study is presented as a pilot investigation, and future randomized controlled trials are needed to confirm its effectiveness. Due to the anonymous data collection, individual pre-test and post-test responses could not be matched, limiting the analysis to descriptive statistics; future studies should adopt a paired design with identifiable data to enable more robust inferential evaluation. We acknowledge that social desirability bias constitutes a limitation of the present study. Future investigations should incorporate more objective measures, such as direct observation, standardized patient interactions, or performance-based assessments.

## 5 Conclusion

In summary, this study demonstrates that the AI-assisted World Café model can effectively promote clinical pharmacology graduate students’ mastery of complex professional knowledge and foster higher-order clinical decision-making competencies. The model successfully integrates intelligent AI assistance with a structured social learning environment, facilitating a pedagogical shift from knowledge transmission toward capacity building. Concurrently, the reliability demonstrated by AI in process evaluation offers a novel perspective for addressing the challenge of scalable formative assessment. While challenges such as information accuracy persist, this human-computer collaborative hybrid teaching model, implemented through carefully designed instructional processes and supervisory mechanisms, presents a promising innovative pathway for preparing future medical professionals for complex healthcare environments.

## Data Availability

The raw data supporting the conclusions of this article will be made available by the authors, without undue reservation.
