# Supplementary material for: Application of the artificial intelligence-assisted World Café teaching model in clinical pharmacology graduate course: a pilot study
Source: Front Public Health. 2026 Apr 24;14:1805521. doi: 10.3389/fpubh.2026.1805521 (PMC13153067; doi:10.3389/fpubh.2026.1805521)
Supplement: Supplementary file 1 [file Data_Sheet_1.pdf]

Rubric for Evaluating AI- Assisted Discussion Records

Dimension	90–100 (Excellent)	75–89 (Good)	60–74 (Fair)	0–59 (Poor)
clinical reasoning and diagnostic logic	Demonstrates comprehensive diagnostic reasoning; correctly identifies core and associated symptoms; provides a well-justified differential diagnosis with clear distinguishing points; logically integrates case information to support the diagnostic conclusion.	Shows sound diagnostic reasoning with minor gaps in differential diagnosis or justification.	Identifies basic diagnostic elements but lacks depth in reasoning or differential analysis.	Limited or incorrect diagnostic reasoning; fails to address key clinical features.
AI-assisted clinical evaluation	Poses precise, mechanism-focused questions; critically evaluates AI responses across multiple dimensions (information verification, logical consistency, identification of blind spots); clearly distinguishes validated content from AI-generated information; demonstrates independent judgment.	Questions are relevant; AI responses are evaluated but with limited critical depth or omission of one appraisal dimension.	Questions are general or vague; appraisal is superficial or primarily descriptive without critical analysis.	Minimal or no engagement with AI outputs; no critical evaluation.
biopsychosocial analysis	Integrates biological, psychological, and social factors into case analysis; identifies relevant etiological factors, treatment implications, and contextual influences; demonstrates holistic understanding.	Addresses all three domains but with limited integration or depth in one domain.	Covers two domains adequately but neglects or superficially addresses the third.	Focuses on a single domain or fails to incorporate biopsychosocial perspective.
teamwork and communication	Record sheet clearly reflects collaborative group work; final conclusions synthesize AI input, inter-group insights, and instructor feedback; ideas are well-organized and clearly communicated.	Group collaboration is evident but synthesis is partial or organization has minor flaws.	Limited evidence of integration or synthesis; conclusions are disjointed or primarily rely on a single source.	No evidence of teamwork or synthesis; responses are incomplete or disorganized.
clinical pharmacology and therapeutic strategy design	Applies pharmacological principles accurately; proposes evidence-based treatment strategies with clear mechanistic rationale; addresses drug selection, dosing considerations, side effect management, and non-pharmacological interventions appropriately.	Treatment strategy is generally appropriate but lacks detailed mechanistic justification or omits some relevant considerations.	Basic treatment suggestions are provided but with limited pharmacological reasoning or incomplete coverage of options.	Incorrect or inappropriate treatment recommendations; fails to demonstrate understanding of pharmacological principles.

1
