# Supplementary material for: Application of the artificial intelligence-assisted World Café teaching model in clinical pharmacology graduate course: a pilot study
Source: Front Public Health. 2026 Apr 24;14:1805521. doi: 10.3389/fpubh.2026.1805521 (PMC13153067; doi:10.3389/fpubh.2026.1805521)
Supplement: Supplementary file 3 [file Table_2.docx]

AI-assisted “World Café” Model in Clinical Pharmacology

Collaborative Learning Handbook

**Group Name:** Group No. _

**Member Roles:** Group Leader (Organizes Discussion):

Chief Questioner (Leads AI Questioning):

Critical Reviewer (Leads AI Response Evaluation):

Recorder (Responsible for Filling this Handbook):

Presenter (Responsible for Final Summary & Presentation):


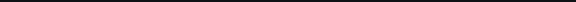


Question 1

Step 1: Formulate human hypotheses · Our starting point

(Before consulting AI, please conduct a group discussion based on the available case materials to form your preliminary judgments.)

| Question 1: List the core and associated symptoms presented by the patient that meet the DSM-5/ICD-11 diagnostic criteria for depression. Which psychiatric disorders or somatic diseases require differential diagnosis, and what are the key differentiating points?  From a biopsychosocial model perspective, analyze the potential etiological factors in this patient. Which pieces of information suggest that this illness may have a “complex” or “treatment-resistant” tendency?  Apart from a detailed psychiatric examination, what additional physical examinations, laboratory tests, and psychological assessments are required to clarify the diagnosis and formulate a treatment plan? Please specify the purpose of each investigation. | | | | |
| --- | --- | --- | --- | --- |
| Which psychiatric disorders or somatic diseases require differential diagnosis? | Key points for differential diagnosis | Which information suggests that this illness may have a “complex” or “treatment-resistant” tendency? | What additional physical examinations, laboratory tests, and psychological assessments are required? | The purpose of each examination |
|  |  |  |  |  |
|  |  |  |  |  |

Step 2: Pose precise questions to AI · Fulcrums for expanding thought

(Please deliberate together and pose 1-2 open-ended, mechanism-focused questions to the AI to help deepen or test your hypotheses.)

**AI Tool Used:**

**Our Questions to AI:**

**Question 1:**

**Question 2:**

**Question 3:**

**Question 4:**

Step 3: Critical appraisal · The wisdom filter

(Please paste or summarize the AI’s responses below and conduct a group evaluation.)

**Summary of Key AI Responses**

**Question 1:**

**Question 2:**

**Question 3:**

**Question 4:**

**Our evaluation and reflection on the AI’s responses:**

**[Information Verification]** Is the information provided by the AI consistent with textbooks/guidelines? Are there any contradictions or innovative points?

**[Logical Consistency]** Can the AI’s explanation fit seamlessly into the evidence chain of this case? Are there any inconsistencies?

**[Identifying Blind Spots]** Are there any omissions in the AI’s response? Did it overlook any key details of this case？

Step 4: Inter-group exchange and integration · The marketplace of ideas

(During the rotation, please record valuable insights gained from other groups.)

**Key viewpoints or insights from other groups**

**Viewpoint 1:**

**Viewpoint 2:**

Step 5: Integration and expression

Our final conclusions (Final version integrating AI input, inter-group information, and instructor feedback：

| Question 1:  List the core and associated symptoms presented by the patient that meet the DSM-5/ICD-11 diagnostic criteria for depression. Which psychiatric disorders or somatic diseases require differential diagnosis? What are the key differentiating points?  From a biopsychosocial model perspective, analyze the potential etiological factors in this patient. Which pieces of information suggest that this illness may have a “complex” or “treatment-resistant” tendency?  Apart from a detailed psychiatric examination, what additional physical examinations, laboratory tests, and psychological assessments are required to clarify the diagnosis and formulate a treatment plan? Please specify the purpose of each investigation. | | | | |
| --- | --- | --- | --- | --- |
| Which psychiatric disorders or somatic illnesses require differential diagnosis? | Key points for differential diagnosis | What information suggests that this condition may have a “complex” or “treatment-resistant” tendency? | What additional physical examinations, laboratory tests, and psychological assessments need to be supplemented? | The purpose of each investigation |
|  |  |  |  |  |
|  |  |  |  |  |

Question 2

Step 1: Formulate human hypotheses · Our starting point

(Before consulting AI, please conduct a group discussion based on the available case materials to form your preliminary judgments.)

**Please provide your preliminary diagnosis (including primary diagnosis and possible comorbidities).**

**What does the concurrent elevation of HAMD and HAMA scores indicate?**

**What implications do the personality trait assessment results have for treatment?**

**Combining the P300 and fNIRS findings, from neurophysiological and brain function perspectives, what pathological mechanisms of depression might this reflect?**

**Despite normal routine blood tests, based on neuroendocrine and neuroplasticity hypotheses, analyze the possible biological basis for this patient’s symptoms (e.g., anhedonia, cognitive slowing).**

**Explain the possible reasons for the patient’s previously poor response and significant side effects to paroxetine.**

Step 2: Pose precise questions to AI · Fulcrums for expanding thought

(Please deliberate together and pose 1-2 open-ended, mechanism-focused questions to the AI to help deepen or test your hypotheses.)

**AI tool used:**

**Our Questions to AI:**

**Question 1:**

**Question 2:**

**Question 3:**

**Question 4:**

Step 3: Critical appraisal · The wisdom filter

(Please paste or summarize the AI’s responses below and conduct a group evaluation.)

**Summary of key AI responses**

**Question 1:**

**Question 2:**

**Question 3:**

**Question 4:：**

**Our evaluation and reflection on the AI’s responses:**

**[Information Verification]** Is the information provided by the AI consistent with textbooks/guidelines? Are there any contradictions or innovative points?

**[Logical Consistency]** Can the AI’s explanation fit seamlessly into the evidence chain of this case? Are there any inconsistencies?

**[Identifying Blind Spots]** Are there any omissions in the AI’s response? Did it overlook any key details of this case?

Step 4: Inter-group exchange and integration · The marketplace of ideas

(During the rotation, please record valuable insights gained from other groups.)

**Key viewpoints or insights from other groups**

**Viewpoint 1:**

**Viewpoint 2**

Step 5: Integration and expression

Our final conclusions (Final version integrating AI input, inter-group information, and instructor feedback):

**Please provide your preliminary diagnosis (including primary diagnosis and possible comorbidities).**

**What does the concurrent elevation of HAMD and HAMA scores indicate?**

**What implications do the personality trait assessment results have for treatment?**

**Combining the P300 and fNIRS findings, from neurophysiological and brain function perspectives, what pathological mechanisms of depression might this reflect?**

**Despite normal routine blood tests, based on neuroendocrine and neuroplasticity hypotheses, analyze the possible biological basis for this patient’s symptoms (e.g., anhedonia, cognitive slowing).**

**Explain the possible reasons for the patient’s previously poor response and significant side effects to paroxetine.**

Question 3

Step 1: Formulate human hypotheses · Our starting point

(Before consulting AI, please conduct a group discussion based on the available case materials to form your preliminary judgments.)

**Considering the patient’s significant anxiety, insomnia, and partial efficacy but poor tolerability of previous SSRI (paroxetine) treatment, which class or specific antidepressant would you choose as the initial treatment? Explain your pharmacological rationale (mechanism of action, receptor targets) and clinical considerations (advantages for core symptom clusters, side effects).**

**If core symptoms (especially lack of motivation and cognitive symptoms) show insufficient improvement after 4-6 weeks of monotherapy, what are the reasonable pharmacological augmentation strategies? Provide examples and explain their mechanisms of action (e.g., atypical antipsychotics, mood stabilizers, certain antidepressant combinations).**

**For your chosen initial regimen, which common side effects require special attention? How would you educate the patient to improve adherence?**

**Given the patient’s issues with poor stress coping, interpersonal conflicts, and low self-worth, which psychotherapy approach (e.g., Cognitive Behavioral Therapy [CBT], Interpersonal Psychotherapy [IPT], Dialectical Behavior Therapy [DBT]) might be most suitable as an initial choice? Why?**

**Physical Therapies: Under what circumstances would you consider introducing repetitive Transcranial Magnetic Stimulation (rTMS) or modified Electroconvulsive Therapy (MECT)? What are their mechanisms of action and potential roles in this case?**

Step 2: Pose precise questions to AI · Fulcrums for expanding thought

(Please deliberate together and pose 1-2 open-ended, mechanism-focused questions to the AI to help deepen or test your hypotheses.)

**AI tool used:**

**Our questions to AI:**

**Question 1:**

**Question 2:**

**Question 3:**

**Question 4:**

Step 3: Critical appraisal · The wisdom filter

(Please paste or summarize the AI’s responses below and conduct a group evaluation.)

Summary of key AI responses

**Question 1:**

**Question 2:**

**Question 3:**

**Question 4:**

**Our evaluation and reflection on the AI’s responses:**

**[Information Verification]** Is the information provided by the AI consistent with textbooks/guidelines? Are there any contradictions or innovative points?

**[Logical Consistency]** Can the AI’s explanation fit seamlessly into the evidence chain of this case? Are there any inconsistencies?

**[Identifying Blind Spots]** Are there any omissions in the AI’s response? Did it overlook any key details of this case?

Step 4: Inter-group exchange and integration · The marketplace of ideas

(During the rotation, please record valuable insights gained from other groups.)

**Key viewpoints or insights from other groups**

**Viewpoint 1:**

**Viewpoint 2:**

Step 5: Integration and expression

Our final conclusions (Final version integrating AI input, inter-group information, and instructor feedback):

**Considering the patient’s significant anxiety, insomnia, and partial efficacy but poor tolerability of previous SSRI (paroxetine) treatment, which class or specific antidepressant would you choose as the initial treatment? Explain your pharmacological rationale (mechanism of action, receptor targets) and clinical considerations (advantages for core symptom clusters, side effects).**

**If core symptoms (especially lack of motivation and cognitive symptoms) show insufficient improvement after 4-6 weeks of monotherapy, what are the reasonable pharmacological augmentation strategies? Provide examples and explain their mechanisms of action (e.g., atypical antipsychotics, mood stabilizers, certain antidepressant combinations).**

**For your chosen initial regimen, which common side effects require special attention? How would you educate the patient to improve adherence?**

**Given the patient’s issues with poor stress coping, interpersonal conflicts, and low self-worth, which psychotherapy approach (e.g., Cognitive Behavioral Therapy [CBT], Interpersonal Psychotherapy [IPT], Dialectical Behavior Therapy [DBT]) might be most suitable as an initial choice? Why?**

**Physical Therapies: Under what circumstances would you consider introducing repetitive Transcranial Magnetic Stimulation (rTMS) or modified Electroconvulsive Therapy (MECT)? What are their mechanisms of action and potential roles in this case?**

**Core Information We Obtained from Textbooks or Other Sources:**

**Our Biggest Takeaway or Newest Understanding from This Session’s Discussion:**
